# Treatment algorithm based on the multivariate survival analyses in patients with advanced hepatocellular carcinoma treated with trans-arterial chemoembolization

**DOI:** 10.1371/journal.pone.0170750

**Published:** 2017-02-07

**Authors:** Hasmukh J. Prajapati, Hyun S. Kim

**Affiliations:** 1 Division of Pediatric Interventional Radiology, Department of Radiology, University of Tennessee Health Science Center, Memphis, TN, United States of America; 2 Division of Interventional Radiology, Department of Radiology and Biomedical Imaging, Yale University, New Haven, CT, United States of America; 3 Yale Cancer Center, Yale University, New Haven, CT, United States of America; Taipei Veterans General Hospital, TAIWAN

## Abstract

**Purpose:**

To develop the treatment algorithm from multivariate survival analyses (MVA) in patients with Barcelona clinic liver cancer (BCLC) C (advanced) Hepatocellular carcinoma (HCC) patients treated with Trans-arterial Chemoembolization (TACE).

**Methods:**

Consecutive unresectable and non-tranplantable patients with advanced HCC, who received DEB TACE were studied. A total of 238 patients (mean age, 62.4yrs) was included in the study. Survivals were analyzed according to different parameters from the time of the 1st DEB TACE. Kaplan Meier and Cox Proportional Hazard model were used for survival analysis. The SS was constructed from MVA and named BCLC C HCC Prognostic (BCHP) staging system (SS).

**Results:**

Overall median survival (OS) was 16.2 months. In HCC patients with venous thrombosis (VT) of large vein [main portal vein (PV), right or left PV, hepatic vein, inferior vena cava] (22.7%) versus small vein (segmental/subsegmental PV) (9.7%) versus no VT had OSs of 6.4 months versus 20 months versus 22.8 months respectively (p<0.001). On MVA, the significant independent prognostic factors (PFs) of survival were CP class, eastern cooperative oncology group (ECOG) performance status (PS), single HCC<5 cm, site of VT, metastases, serum creatinine and serum alpha-feto protein. Based on these PFs, the BCHP staging system was constructed. The OSs of stages I, II and III were 28.4 months, 11.8 months and 2.4 months accordingly (p<0.001). The treatment plan was proposed according to the different stages.

**Conclusion:**

On MVA of patients with advanced HCC treated with TACE, significant independent prognostic factors (PFs) of survival were CP class, ECOG PS, single HCC<5 cm or others, site of VT, metastases, serum creatinine and serum alpha-feto protein. New BCHP SS was proposed based on MVA data to identify the suitable advanced HCC patients for TACE treatments.

## Introduction

Hepatocellular carcinoma (HCC) is the most common primary malignancy of the liver, accounts for the sixth most common malignancy worldwide [1] and the third most common cause of cancer-related death globally behind only lung and stomach cancers [2]. Because most patients present with advanced disease, curative surgical resection is an option for less than 20% of the patients [3] and their available treatment options are different locoregional therapies. Among these unresectable HCC patients, the patients with advanced stage HCC have limited treatment options [4–6]. Barcelona-Clinic Liver Cancer (BCLC) staging treatment algorithm of HCC is widely used in Western countries. According to the BCLC treatment algorithm, the sorafenib has been the proposed care for the HCC patients with BCLC C stage [6]. However, several doxorubicin drug eluting beads trans-arterial chemoembolization (DEB TACE) studies have also shown survival benefits in patients with advanced stage HCC [7, 8]. The two recent prospective studies of DEB TACE with sorafenib have shown promising efficacy in patients with advanced stage HCC [9, 10]. As most HCC patients present with advanced disease, it is important to identify the independent variables of improved survival after TACE and to identify the BCLC C (advanced stage) HCC patients who can get maximum benefit from TACE. This information can be very useful to select the TACE therapy for the correct patient with advanced stage HCC.

The staging of HCC is challenging because the most of the patients with HCC have underlying liver disease, which can have a significant impact on the prognosis apart from the biology of the tumor. Despite numerous validations of different staging systems, there is no single staging system that could be called the “standard” for classifying HCC. The purpose of the present study is to investigate the independent prognostic factors of survival after TACE in patients with advanced HCC. We developed a new prognostic staging system named as BCLC C HCC Prognostic (BCHP) staging system and proposed a treatment algorithm for the advanced HCC patients.

## Materials and methods

This is a single institution prospective study with the patient’s written consent, approved by the Emory University Hospital Institutional Review Board (IRB) and is Health Insurance Portability and Accountability Act (HIPAA) compliant. The consents were recorded electronically and procedure was approved by the IRB.

### Patient selection

**Fig 1:**
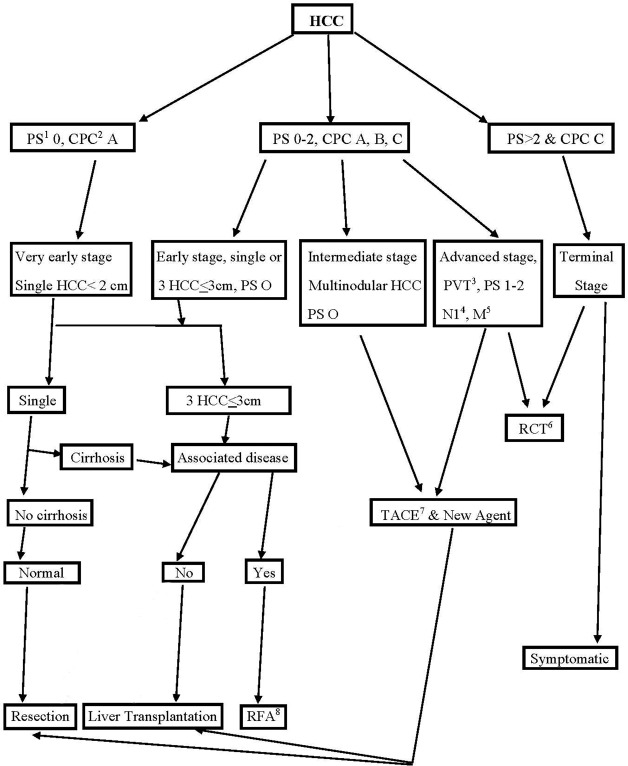
Interventional Oncology guideline of our institute for patients with HCC based on BCLC proposal. (1) Perfomance status, (2) Child Pugh class, (3) Portal vein thrombosis, (4) Nodal metastases at porta hepatis, (5) Metastases, (6) Randomized controlled trial, (7) Doxorubicin drug eluting beads trans-arterial chemoembolization, (8) Radiofrequency ablation.

Between December 2005 and March 2013, 420 consecutive HCC patients were treated with DEB TACE. The patients with advanced HCC, Child Pugh score <10, eastern cooperative oncology group (ECOG) performance status (PS) < 3 and HCC not amenable for radiofrequency ablation were treated with DEB TACE and included in the studies. Our institutional Interventional oncology guideline for the patients with HCC is shown in Fig 1. The patients who received treatment with sorafenib were also included in the study. Patients were excluded from this analysis if they received bland embolization or radioembolization treatment. The patients who underwent orthotopic liver transplantation or surgical liver resection or radiofrequency ablation were also excluded from the study. A total of 238 patients (mean age, 62.4yrs) met the inclusion criteria and was included in the study. The patients were staged with advanced (BCLC C) disease on the basis of Eastern Cooperative Oncology Group performance status (ECOG PS) in 52.52% of cases, portal vein invasion or PVT only in 28.57% of cases, metastasis only in 10.6% of cases, and both metastasis and PVT in 7.8% of cases (Table 1). All patients had contrast enhanced triphasic CT scan or MRI of abdomen studies before the consultation. The ECOG PS and serum alpha-feto protein (AFP) level of each patient were assessed before the TACE procedure. The functional liver status was determined by using the Child-Pugh criteria.

**Table 1:** Factors Responsible for Staging of Advanced Stage (BCLCC) in Patients with HCC.

ECOG PS	No. (%)	No Mets or PVT, No. (%)	Only PVT, No. (%)	Only Mets, No. (%)	PVT and Mets, No. (%)
0	45 (18.9%)	0	26 (10.9%)	11 (4.62%)	8 (3.36%)
1	155 (65.1%)	102 (42.85%)	23 (9.66%)	10 (4.2%)	9 (3.78%)
2	38 (16.0%)	23 (9.66%)	9 (3.78%)	4 (1.68%)	2 (0.84%)
Total	238	125 (52.52%)	68 (28.57%)	26 (10.6%)	19 (7.98%)

### Study objective

The primary objective of the study was to investigate the independent prognostic factors of survival, to construct a prognostic staging system and treatment algorithm in patients with BCLC C HCC after DEB TACE. Secondary aims were to compare overall survival in subgroups based on different imaging and laboratory findings, the presence or absence of extra-hepatic metastasis and/or portal vein thrombosis, and staging systems, in patients with BCLC C HCC treated with DEB TACE. We have also assessed the recent new staging system [11] from Hong Kong Liver Cancer (HKLC) group.

### Imaging of HCC

The AASLD (American association for the study of liver disease)—JNCI (Journal of the National Cancer Institute) guidelines [4] were used to diagnose HCC. In this study, 92% of the patients were diagnosed by MRI and 8% of patients were diagnosed by histopathology examination after biopsy. The imaging features of HCC in all patients were interpreted by two radiologists with 12 and 10 years of experience in interpreting MR imaging of the abdomen and pelvis. The MRI imaging protocol of our institute is similar to the one of the published studies [12].

### TACE procedure and follow up

Six hundred two DEB TACE procedures performed were performed in 238 patients (mean of 2.53± SD 1.8, range: 1 to 11). All of the therapies were performed under moderate sedation and local analgesia. Dosages of 0.5–4 mg midazolam (Novaplus, Hospira Inc., Lake Forest, IL, USA) and 50–400 mcg fentanyl (Sublimaze, Baxter, Deerfield, IL, USA) were administered intravenously during TACE for moderate sedation.

After ultrasound-guided femoral artery cannulation, each first-time procedure was initiated with diagnostic celiac and superior mesenteric angiograms with a 5F Simmons 1 (Terumo, Somerset, NJ, USA) catheter to outline the anatomy, delineate the tumor(s), and identify the portal vein. The third or fourth order branches of feeding vessels supplying the HCC were catheterized with a 2.1 F microcatheter (STC Renegade Hi-Flo; Boston Scientific, Natick, MA, USA). Then, the tumors were embolized with a slow fluoroscopy-guided injection of iodinated contrast mixed 300–500 and 500–700 μm LC beads (9%) or with 100–300 μm LC beads (91%) impregnated with 50 mg of doxorubicin in each vial. The endpoint for treatment included the administration of the 100 mg of doxorubicin or sluggish flow in the segmental branches of the hepatic artery to the region of the tumor, without an effect on the flow in the main or lobar hepatic artery.

Patients were brought back in 4 weeks for a repeat session who had large tumors of more than 5 cm or multifocal disease and the remainder of the patients were followed up in the clinic in 4 weeks with liver function tests and an MRI of the liver. The patients with complete response after treatment were followed in clinic every 6 months with MRI of the liver and liver function tests.

### Statistical analysis

Patients were stratified on the basis of different demographic, staging systems, imaging and laboratory parameters. Survival was calculated from the time of 1^st^ DEB TACE therapy. The Kaplan–Meier method with the log rank test was used for univariate analysis and Cox proportional model was used to perform multivariate analysis. The detailed pre-treatment imaging findings were included in the multivariate analyses (MVA). A p-value of 0.05 was held as significant. SPSS software, version 21.0 (IBM, Somers, NY) was used to perform the statistical analyses.

## Results

### Patient population

Detailed patient demographics, tumor characteristics and staging at the time of initial presentation with corresponding median survivals are shown in Table 2. The mean size of the index tumor was 5.6 cm (SD ±3.85 cm). Portal vein thrombosis or invasion (PVT) was present in 32.3% of patients and extra-hepatic metastases were present in 18.5% of the patients at the time of initial presentation. Out of the 44 patients (18.5%) with extra-hepatic metastases, the common locations of extra-hepatic metastases were abdominal lymph nodes in 43.2% of cases, lungs in 22.7% of cases, and the adrenal gland in 25% of cases.

**Table 2:** Demographics, etiology, staging, liver disease, imaging characteristics and laboratory findings of the patients with advanced (BCLC C) HCC before 1^st^ TACE treatment and corresponding survivals from 1st TACE.

Parameters	Values *N*	Median Survival (months) (95% CI^@^)	*P* value from log rank test
**[I] DEMOGRAPHICS**			
**Total number of BCLC C HCC patients**	238	16.2 (11.7, 20.7)	
**Age at Diagnosis (yrs)**			
Mean(SD)	62.4 (10.9)		
**Gender**			
Male	179 (75.2%)	15.8 (10.7, 20.9)	0.77
Female	59 (24.8%)	17.7 (7, 28.5)	
**Ethnicity**			
Caucasian	156 (65.5%)	19.5 (12.9, 26)	0.08
African American	53 (22.3%)	17.8 (8.3, 27.3)	
Others	29 (12.2%)	13 (8.8, 17.1)	
**[II] ETIOLOGY**			
**1.** Hepatitis C	146 (61.3%)	15.3 (9.6, 21.1)	0.1
**2.** Hepatitis B	19 (8.0%)	8.8 (5.1, 12.5)	
**3.** Alcohol	17 (7.2%)	30.8 (1, 61.7)	
**4.** Cryptogenic cirrhosis	33 (13.8%)	19.5 (1.3, 37.7)	
**5.** Other causes of chronic liver disease	9 (3.8%)	20.8 (6.5, 35.1)	
**6.** No cirrhosis	14 (5.9%)	11.8 (3.8, 16.2)	
**[III] STAGINGS**			
**Okuda staging**			
I	112 (47.1%)	25.6 (19.5, 31.7)	<0.0001
II	126 (52.9%)	10.1 (8.2, 12.0)	
**Cancer of the Italian Liver Program staging**			
Early	31 (13.0%)	34.5 (28.1, 40.8)	<0.0001
Intermediate	188 (79.0%)	13.6 (9.5 17.7)	
Advanced	19 (8.0%)	4.5 (3.6, 5.4)	
**ECOG Perfomance status**			
0	45 (18.9%)	20.0 (6.5, 33.5)	0.005
1	155 (65.1%)	17.7 (12.7, 22.8)	
>1	38 (16.0%)	7.3 (3.5, 11.1)	
**Hong Kong Liver Cancer Staging System**			
**1.** IIa	74 (31.1%)	28 (16.3, 39.7)	<0.0001
**2.** IIb	27 (11.3%)	24.2 (14.3, 34.1)	
**3.** IIIa	19 (8.0%)	9.4 (8.1, 21.3)	
**4.** IIIb	25 (10.5%)	12.9 (10.1, 15.8)	
**5.** IVa	37 (15.5%)	9.3 (7.3, 11.3)	
**6.** IVb	18 (7.6%)	11.3 (7.5, 15)	
**7.** Va	18 (7.6%)	15.1 (0.7, 29.4)	
**8.** Vb	20 (8.4%)	6.2 (2.5, 10)	
**[IV] ADJUVANT SORAFENIB THERAPY**			
Present	48 (20.2%)	17.2 (10.2, 21.5)	0.92
Absent	190 (79.8%)	15.8 (6.2, 28.2)	
**[V] LIVER DISEASE**			
**Child-Pugh class**			
A	132 (55.5%)	22.3 (16.2, 28.3)	0.004
B	106 (44.5%)	10.9 (7.7, 14.1)	
**Cirrhosis**			
Present	224 (94.1%)	16.2 (11.7 20.7)	0.83
Absent	14 (5.9%)	11.8 (3.8, 16.2)	
**Ascites**			
Absent	177 (74.4%)	21.0 (15.5, 26.5)	0.001
Present	61 (25.6%)	9.5 (7.7, 11.3)	
**Portal hypertension**			
Absent	85 (35.7%)	19.9 (10.3, 29.4)	0.005
Present	153 (64.3%)	12.9 (8.3, 17.5)	
**[VI] TUMOR MORPHOLOGY**			
**Tumor locations**			
Unilobar	164 (68.9%)	19.5 (13.4, 25.5)	0.057
Bilobar	74 (31.1%)	11.1 (6.5, 15.7)	
**Type of HCC on Imaging**			
**1.** Typical feature of HCC	183 (76.9%)	17.2 (12.2, 22.1)	<0.0001
**2.** Blood products containing HCC	7 (2.9%)	9.4 (8.4, 10.4)	
**3.** Fat containing HCC	12 (5.1%)	34 (28.4, 39.5)	
**4.** Fibrolamellar HCC	2 (0.8%)	15.3	
**5.** Infiltrative HCC	29 (12.2%)	4.5 (2.3, 6.8)	
**6.** Mixed features of HCC and intrahepatic cholangiocarcinoma	5 (2.1%)	17.7 (12.7, 21.7)	
**Number of tumors**			
Solitary	114 (47.9%)	22.9 (18.5 27.4)	0.018
Two HCCs	54 (22.7%)	11.3 (6.7, 15.8)	
>2 HCCs	70 (29.4%)	11.8 (7.1, 16.6)	
**Size of index tumor**			
<4cm	102 (42.8%)	21.7 (17.1, 26.4)	0.12
4–8 cm	88 (37.0%)	15.9 (8.6, 23.1)	
>8 cm	48 (20.2%)	10.3 (6.7, 13.7)	
**Portal vein thrombosis**			
Present	77 (32.3%)	10.1 (6.7, 13.5)	0.002
Absent	161 (67.7%)	20 (14.3, 25.7)	
**Extra-hepatic metastasis**			
Present	44 (18.5%)	8.8 (5.7, 11.9)	<0.0001
Absent	194 (81.5%)	19.9 (15.1, 24.7)	
**[VII] LABORATORY FINDINGS**			
**Total serum bilirubin level (mg/dl)**			
< 2	206 (86.6%)	18.8 (14.4, 23.2)	0.004
2 to 3	25 (10.5%)	7.7 (4.3, 11.3)	
>3	7 (2.9%)	10.1 (0.4, 22)	
**Serum Albumin level (mg/dl)**			
>3.5	64 (26.9%)	28 (17.2, 38.8)	<0.0001
2.8 to 3.5	123 (51.7%)	16.2 (9.9, 22.5)	
<2.8	51 (21.4%)	8.9 (4.0, 13.8)	
**Serum Creatinine (mg/dl)**			
<1.2	196 (82.4%)	17.7 (11.5, 24)	0.04
>1.2	42 (17.6%)	11.8 (8.7, 15)	
**International normalized ratio (INR)**			
<1.5	224 (94.1%)	17.7 (12.9, 22.6)	0.02
>1.5	14 (5.9%)	6.7 (1.6, 11.8)	
**Serum Alpha Fetoprotein level(ng/dl)**			
<400	170 (71.4%)	21.7 (16.6, 26.8)	<0.0001
>400	68 (28.6%)	6.8 (5.4, 8.2)	

CI@—Confidence interval.

### Survival analysis

The overall median survival was 16.2 months from the time of 1^st^ DEB TACE. The univariate survival analyses were performed for different categories as shown in Table 2. The median survivals according to Child Pugh class A (55.4%) and B (44.6%) were 22.3 months and 10.9 months respectively (*p* = 0.004). Median survivals in HCC patients without PVT and with PVT treated with DEB TACE were 20 and 10.1 months (*p* = 0.002), respectively. The survivals were significantly different when the PVT was stratified according to the site of the venous thrombosis (*p*<0.001). In patients with advanced HCC, 22.7% of the patients (*n* = 54) had venous thrombosis of the large vein (main portal vein, right or left portal vein, hepatic vein, inferior vena cava), 9.7% of the HCC patients had thrombosis of small veins (segmental/subsegmental PV) and their corresponding overall median survivals were 6.4 months and 20 months respectively (*p*<0.001).

The median survivals in HCC patients with and without extra-hepatic metastasis were 8.8 and 19.9 months (*p*<0.0001) respectively. The patients with infiltrative HCC had the poorest survival of 4.5 months. The patients with fat containing HCC on MRI has the best overall median survivals of 34 months among others (*p*<0.0001, Table 2).

#### Multivariate analyses

The hazard ratios (HRs) were calculated by multivariate analyses performed by Cox proportional model. HCC imaging characteristics were accounted in multivariate analysis. On multivariate analysis, the significant independent prognostic factors of survival were Child Pugh class, ECOG PS, the presence of single HCC (<5 cm versus others who did not have single HCC<5 cm in size), the site of the PVT (large versus small veins), the presence of extra-hepatic metastasis, serum creatinine (≥1.2 mg/dl), and AFP (>400 ng/dl) (Table 3).

**Table 3:** Multivariate survival analyses with COX model adjusting for all important covariates in a cohort of 238 patients with advanced HCC.

Variables	P value	Hazard ratio (HR)	95.0% CI* for Exp(B)	
			Lower	Upper
**Child Pugh Class**				
A	0.003	Reference		
B		1.8	1.2	2.5
**Eastern cooperative oncology group (ECOG) Performance status (PS)**				
ECOG PS 0	<0.0001	Reference		
ECOG PS 1	0.04	1.7	1.02	2.9
ECOG PS > 1	<0.0001	3.5	1.9	6.6
**Single HCC <5 cm**				
No	0.01	Reference		
Yes		0.6	0.4	0.9
**Extent and site of the portal or hepatic vein invasion**				
No portal vein invasion	0.010	Reference		
Small vein invasion	0.04	1.4	1.01	2.2
Large vein invasion	0.002	2.02	1.3	3.2
**Extra-hepatic metastasis**				
Absent	0.04	Reference		
Present		1.6	1.02	2.5
**Serum Alpha Fetoprotein level > 400 ng/dl**				
No	<0.0001	Reference		
Yes		2.1	1.4	3.1
**Serum Creatinine (mg/dl)** **≥1.2**				
No	0.009	Reference		
Yes		1.8	1.2	2.7

CI* = Confidence Interval.

### Development of the prognostic classification scheme and survival analysis

**Fig 2:**
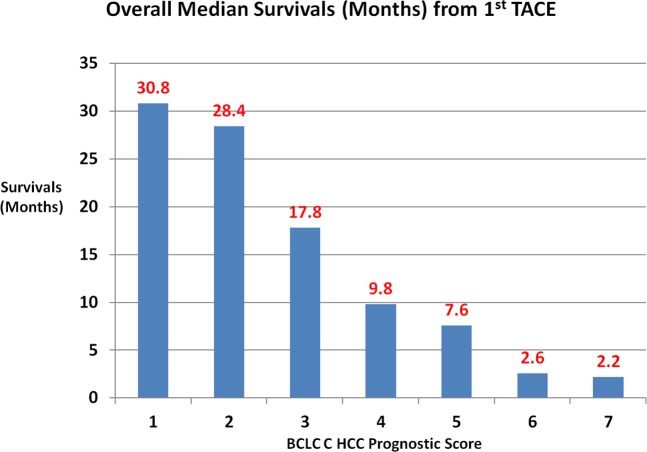
The overall median survival, according to the BCHP scores.

**Fig 3:**
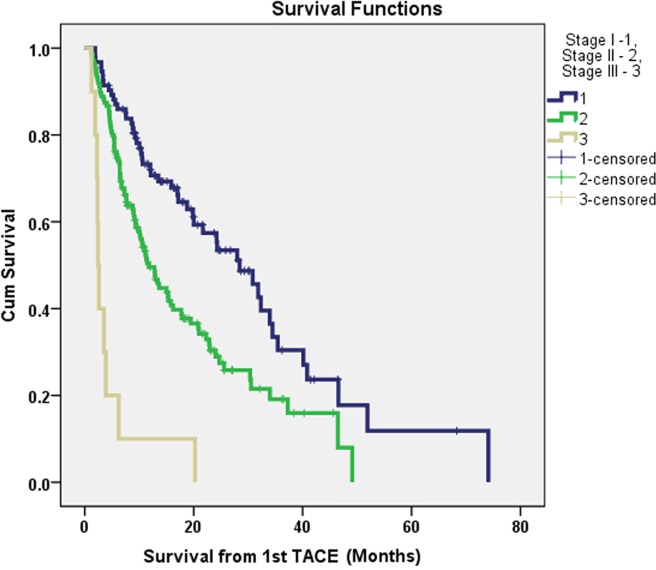
The Kaplan Meier survival graph demonstrating the survival difference after TACE treatments in HCC patients according to BCHP staging.

The prognostic classification scheme was constructed from the significant independent prognostic factors of survival from the multivariate Cox proportional hazards regression model. The reference category of the each prognostic factor was assigned a value of zero. The hazard ratios of the all the independent prognostic factors of survival were used to construct the scoring system for the prognostic staging. The Child Pugh class, ECOG PS, the presence of single HCC (<5 cm versus others), the site of the PVT (large versus small veins), the presence of extra-hepatic metastasis, serum creatinine (≥1.2 mg/dl), and AFP (>400 ng/dl) were selected in building the prognostic staging system. The scoring system was tested with univariate survival analysis. Based on the different survival analyses of the different scores and clinical judgment with, staging system was constructed and named as BCLC C HCC Prognostic (BCHP) staging system. The proposed BCHP staging system is shown in the Table 4. The performance of this prognostic staging system was assessed by cumulative survival analysis. The overall survival analysis according to the different BCHP score is shown in Fig 2. The overall median survivals of stage I (n = 93, 39.1%), II (n = 135, 56.7%) and III (n = 10, 4.2%) were 28.4 months, 11.8 months and 2.4 months accordingly (*p*<0.0001, Tables 4–6 and Fig 3).

**Table 4:** BCLC C HCC Prognostic (BCHP) staging system for the patients with advanced HCC treat with TACE.

No	Variables	Score 0	Score 1	Score 2
1	Child Pugh Class	A	B	
2	ECOG PS	0	1	>1
3	Single HCC <5 cm	Yes	No	
4	Venous Thrombosis	No	Small vein invasion	Large vein invasion
5	Metastases	No	Yes	
6	S.Creatinine	<1.2 mg/dl	≥1.2 mg/dl	
7	S.AFP	<400 ng/dl	≥400ng/dl	

Stage I–score 0 to 2.

Stage II—score 3 to 5.

Stage III—score >5.

**Table 5:** The overall median survivals in patients with advanced HCC treated with TACE according to BCLC C HCC Prognostic (BCHP) score.

BCHP score	Value (%)	Median Survival (months) (95% CI^@^)	P value from log rank test
1	36 (15.2)	30.8 (19.4, 42.3)	0.0001
2	57 (23.9)	28.4 (13.7, 43.2)	
3	73 (30.7)	17.8 (11, 24.6)	
4	39 (16.4)	9.8 (6.9, 12.7)	
5	23 (9.7)	7.6 (5.8, 9.4)	
6	8 (3.4)	2.6 (1, 4.2)	
7	2 (0.8)	2.2	
Overall	238 (100)	16.2 (11.7, 20.7)	

**Table 6:** The overall survivals of different BCLC C HCC Prognostic (BCHP) stages in patients with advanced HCC treated with TACE.

Stage	Total score	*N* (%)	OS* (months)	*P* value
I	0–2	93 (39.1)	28.4	<0.001
II	3–5	135 (56.7)	11.8	
III	>5	10 (4.2)	2.4	

OS*–Overall survival

### Treatment recommendation for the BCLC advanced stage HCC from BCHP staging

**Fig 4:**
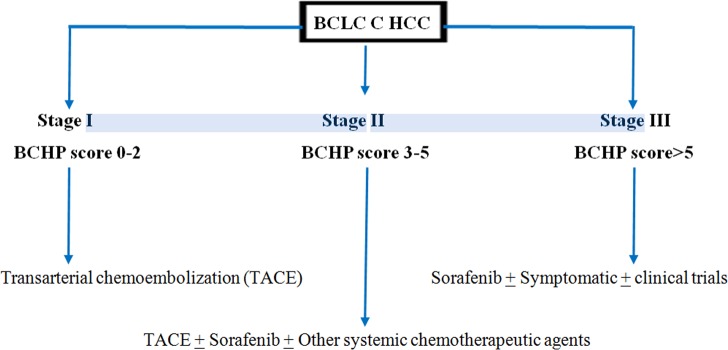
BCLC C HCC Prognostic (BCHP) staging and proposed treatment plan in patients with BCLC C HCC treated with TACE and not amenable for ablative treatments.

The whole cohort is of patients with advanced (BCLC C) unresectable HCC. The radiofrequency ablation was not feasible in these patients. The survivals of the advanced HCC patients, according to the BCHP stages, is shown in Table 6. The advanced HCC patients with BCHP stage I had the highest survival of 28.4 months and most benefited from the DEB TACE treatment. Therefore, the advanced unresectable HCC patients, who are not candidates for ablative therapy, should receive the conventional or DEB TACE. The BCLC C patients with stage III had the poorest survival of 2.4 months and did not get the survival advantage from the DEB TACE treatment. The TACE should not be performed in the stage III patients. The systemic chemotherapy or clinical trials can be useful in these patients. The stage II patients had the overall survival of 11.8 months. The stage II patients may get benefit from the combination of the DEB TACE and systemic chemotherapy. The proposed treatment algorithm in advanced HCC patients is shown in the Fig 4.

## Discussion

As compared to other cancers, the treatment options and prognosis of the HCC not only depend on the morphology of the tumor but also on the extent on impaired liver function. There is also heterogeneity in different viral and metabolic conditions at the root of the HCC. Identification and appropriate quantification of the relevant prognostic factors are very crucial to leading the development of the staging systems. This study investigated the multiple imaging, clinical and serum examination variables and the BCHP staging system was proposed based on the MVA.

Most of the literature addressing the prognostic factors following TACE has been related to conventional TACE (cTACE) [13–20]. Child–Pugh class, tumor burden, and PV thrombosis are considered to be the main prognostic factors for survival following cTACE [13–20]. Several similar studies also addresses the prognostic factors after DEB TACE [8, 21–25]. The initial dedicated study, on prognostic factors in patients with HCC treated with DEB TACE, by Dhanasekaran R et al [25] included fifty patients (39 women and 11 men) with a median age of 57.5 years (range 28–91 years). The tumor size and PVT, which were among the prognostic factors for cTACE studies, were not found to influence survival after treatment with DEB in this study [25]. According to the authors [25], super-selective embolization techniques may partially explain the result in the patients with PVT. This finding was supported in our study. Additionally, the HCC patient with poor kidney function (serum creatinine ≥ 1.2 mg/dl) was found the independent risk factor for death after TACE in our study. This finding was indirectly supported by several studies where the Model for end-stage liver disease (MELD) was the prognostic factor of survival [25, 26]. In this study, the patients with the infiltrative type HCC had the poorest median survival of 3.8 months. This finding is also supported by the study of Sellers M et al [21]. The patients with fat containing HCC had the best median survival of 34 months. Cytoplasmic fat is frequently present in the well-differentiated HCC and suggests the reason of the higher survival in fat containing HCC [27].

Recently, Hong Kong Liver Cancer (HKLC) group proposed a staging system which concluded that HKCL provided better prognostic differentiation than BCLC [11]. We have compared the analysis of urivariate survival data of BCHP staging system with HKCL staging system. The majority of the patients in the study has hepatitis C disease compared to hepatitis B disease in the HKCL study, so this staging system is compatible with the western (American) population. The patients with hepatitis C disease have significant cirrhosis with impaired liver function, whereas the patients with hepatitis B disease with HCC, in general have preserved liver function. There are several advantages of the BCHP staging system over HKLC staging system. The BCHP staging system gives quantitative numbers and is simple to calculate. The HKCL appears complicated to apply and has multiple stages of treatment. The BCHP staging system better identifies patients with extremely poor prognosis. It also identifies the BCLC C patients who can get the benefit of the DEB TACE. The portal vein invasion with HCC is classified as BCLC stage C and suggested treatment is only systemic chemotherapy. Here, it is important to note that BCLC staging was developed more than 10 years ago. In the last 10 years, there are further refinement in the catheter locoregional therapy and techniques. For example, many DEB TACE studies have also shown significantly improved survival in patients with advanced stage HCC [7, 8, 28]. In addition, our proposed staging system has been constructed out of clinical results for Western patients, as compared to the HKCL out of Asian patients with etiologies pertinent to each region.

In this study, the whole cohort is of patients with BCLC C HCC. The BCLC C patients with BCHP stage I benefited from the DEB TACE treatment. The BCLC C patients with BCHP stage III had the poorest survival of 2.4 months and did not get the survival advantage from the DEB TACE treatment. Therefore, it is suggested that the TACE should not be performed on the BCHP stage III HCC patients. The stage BCLC C disease HCC patients with BCHP stage II had the overall survival of 11.8 months and these patients may get benefit from the combination of the TACE and systemic therapy. In this study, the patients who received adjuvant therapy with sorafenib had higher survivals (17.2 months) as compared to the patients who did not receive sorafenib therapy (15.8 months). However, the survival advantage was not statistically significant (*p* = 0.92).

Our study has several limitations. First, the study is a single institution nonrandomized study, so selection bias and late look bias may be inherent. Second, patients who were treated with previous sorafenib treatments were also included in this study, so outcomes after this TACE may be confounded. However, the previous therapies with sorafenib comprised of small volume and did not significantly affect survival in univariate or multivariate analysis. Therefore, we believe that survival advantage in this study is largely from the effect of DEB TACE therapy. Third, this study did not include the advanced HCC patients who received the ablative therapy. So, this study relates to those patients who are not candidates for the ablative therapy. Fourth, the sample size to compare with HKLC staging system is relatively small. So, a validation in a large sample size is required to determine if these staging systems can be accurately used to stratify patients in clinical trials and to help direct patient care.

In summary, the independent prognostic factors for survival following TACE were Child Pugh class, single HCC size <5cm, site of the PVT, the presence of extra-hepatic metastasis, serum creatinine, ECOG PS and serum AFP. The new prognostic staging system was constructed, comprised of the independent factors, established and named as BCHP. The BCHP staging system is simple to use and it identifies the advanced HCC patients who can get maximum benefit from chemoembolization treatment. The advanced HCC patients with BCHP stage I and II may benefit from TACE.
